# Service suspension for mental disorders in armed forces draftees in the Penghu area

**DOI:** 10.1186/1471-244X-12-46

**Published:** 2012-05-23

**Authors:** Wei-Chen Chuang, Chin-Han Kao, Chih-Kang Chen, Chia-He Peng, Wu-Hsi Wang

**Affiliations:** 1 Department of Psychiatry, Tri-Service General Hospital, Taipei, Taiwan; 2 Department of Psychiatry, Beitou Armed Forces Hospital, Taipei, Taiwan; 3 Department of Psychiatry, Penghu branch, Tri-Service General Hospital, Penghu, Taiwan; 4 Department of Psychiatry, Penghu Hospital of Executive Yuan, Penghu, Taiwan

**Keywords:** Military draftees, Service suspension, Military readiness, Psychiatric discharge

## Abstract

**Background:**

It is important to monitor draftees for mental disorders before or at an early stage of military service. The aim of this study was to characterize the draftees who were suspended from service for mental disorders among draftees in a high readiness military zone in the Taiwan Strait.

**Method:**

A total of 152 draftees consulted the outpatient service of the Department of Psychiatry at Penghu branch, Tri-Service General Hospital in Taiwan during the period between August 2004 and July 2008, and whose severity of mental disorder fit the criteria for service suspension were recruited as the study group (SG). Draftees who had adjusted normally were the control group (CG).

**Results:**

The major causes for suspension were major depressive disorders and personality disorders. In the study group, the number of draftees seeking psychiatric outpatient treatment increased from 49.3% before service to 100% during service. In addition, higher rates of suicidal ideation, suicide plans, attempted suicide, and homicidal ideation were found in the study group than in the control group. The percentages of draftees who were unwilling to serve and absent without official leave (AWOL) during military service in Penghu were also significantly higher in the study group than in the control group.

**Conclusions:**

Based on the characteristics of the draftees who were suspended from service for mental disorders, psychological factors such as suicidal ideation, suicide attempts and adjustment disorders should be surveyed and monitored before the draft and at an early stage of military service.

## Background

Many studies have been done to determine the mental health needs of military personnel. A study by Sareen et al. in 2007 in the United States found that 23.2% of active duty personnel perceived a need for mental health services. Deployment to combat operations and witnessing atrocities were associated with an increased prevalence of mental disorder and a perceived need for care. Deployment to peacekeeping operations was not associated with an increase [1]. One study that was conducted just before military operations began in Iraq and Afghanistan found that at least 6% of all U.S. military services members on active duty received treatment for a mental disorder each year [2]. Another study suggested that as many as 9% of soldiers may be at risk for mental disorders before combat deployment and as many as 11 to 17% may be at risk for such disorders 3 to 4 months after their return [3].

Conscription has been employed in Taiwan for more than 60 years and draftees may serve in a high military alert zone such as Penghu. The Penghu islands in the Taiwan Strait play a critical role in national defense and trade for Taiwan. Perceived threats of invasion and political instability create heightened tension and stress for the military personnel stationed there. At present, there is no effective screening for mental disorders prior to assignment. However, draftees who fail to adjust to this duty may apply for service suspension due to mental disorder during their military service. To do so, they must meet the criteria for mental disorders specified in the Regular Servicemen Disability Criteria established by the Medical Affairs Bureau, Ministry of National Defense. These include psychoses (schizophrenia, bipolar affective mood disorders, delusional disorders, schizoaffective disorders, and psychiatric disorder not otherwise classified), major depressive disorder, anxiety disorders, intellectual insufficiency, personality disorders, gender identity disorder, and psychotic disorders due to general physical condition as defined by DSM IV-TR (Diagnostic and Statistical Manual 4th edition-Text Revision, American Psychiatric Association, Washington, DC).

This study was the first attempt to investigate the characteristics of draftees who were suspended from military service due to mental disorders in a high readiness military zone in Taiwan. This case control study compared draftees who failed to adjust to military service in this tense environment with those who made a normal adjustment. The purpose of this study was to determine the characteristics of these service suspended draftees in order to survey for mental disorders before the draft and to monitor for adjustment disorder at an early stage of military service.

## Methods

### Participants

This study was approved by the Institutional Review Board of Tri-Service General Hospital (No. 093-04-125) and informed, written consent was signed by all participants.

A total of 213 draftees in the Penghu Area were self-referred to the psychiatric outpatient service at the Penghu branch, Tri-Service General Hospital between August 2004 and July 2008. A total of 152 draftees met the criteria for service suspension due to “mental disorders” based on the definition of DSM IV-TR after several months of observation and repeated diagnoses. These then became the study group (SG). The control group (CG) consisted of 152 draftees matched from the same draft round and serving in the same units as the study group. The frequency matching was done by the researchers based on age and marital status and not by officers in the units. The officers in each military units provided a list of matched draftees with no apparent mental disorder. The member of the control group was then randomly selected by the researchers from the list of matched draftees according to the frequency of age and marital status among study group. Each identified member of the control group was followed for at least 6 months of normal adjustment and none became a study subject. The total number of troops stationed on Penghu is a military secret; however, we estimate that approximately 20,000 rotate through for periods of 10 months to a year. This would suggest that the prevalence of severe mental disorders was less than 0.2%.

Draftees in both groups completed the Basic Data Survey. The questions were developed by two senior psychiatrists and included: personal data, family data, family history, military history, history of seeking mental health services, history of alcohol and substance abuse, suicidal ideation, plans and attempts, willingness for military service, willingness to serve on the Penghu offshore islands, and willingness to be absent without official leave (AWOL). “Willingness” was measured on a five point Likert scale ranging from unwilling (0) to very high willingness (5). Family attitudes about draftees’ mental disorders were determined during visitation or by telephone inquiry.

### Statistics and analysis

Comparisons between groups were tested using independent two sample t tests for normally distributed continuous variables and Chi-square/Fisher’s exact tests for categorical variables. Continuous variables were displayed as mean ± standard deviation (S.D.) and categorical data were represented by number (n) and percentage (%). The Mann–Whitney-*U* test was applied for skewed variables. All statistic assessments were two-tailed and considered significant at the 0.05 level. Statistic analyses were performed using SPSS 15.0 statistics software (SPSS Inc, Chicago, IL).

## Results

One hundred and fifty-two subjects meeting the criteria for service suspension due to “mental disorders” were selected as the study group. Matched draftees who had adjusted normally to the high tension environment made up the control group. All subjects were male.

Major depressive disorder was found in 43.4% of the study group and personality disorders in 19.1%. The detailed distribution of DSM-IV-TR diagnoses is shown in Table 1.

**Table 1:** Distribution of DSM-IV-TR diagnoses in the study group (n = 152)

**Diagnoses**	**Case group (n = 152)**
**Axis I diagnoses**	
Major depressive disorder	
Single episode only	32 (21.1%)
Recurrent episode	34 (22.4%)
Bipolar affective disorder (all were bipolar II disorder)	23 (15.1%)
Schizophrenia	5 (3.3%)
Schizoaffective disorder (all were bipolar type)	3 (2.0%)
Anxiety disorder	
OCD (obsessive compulsive disorder)	3 (2.0%)
GAD (generalized anxiety disorder)	3 (2.0%)
**Axis II diagnoses**	
Borderline intellectual functioning (FsIQ = 70-84)	13 (8.6%)
Mild mental retardation [FsIQ = 50(or 55)-69]	7 (4.6%)
Moderate, severe or profound MR(mental retardation)	0
Personality disorder	
Antisocial PD only	19 (12.5%)
Borderline PD only	7 (4.6%)
Both antisocial and borderline PD	3 (2%)

Table 2 compares demographics between the study and control groups. There were no significant differences in age, education, marital status, service distribution, and rank distribution between the two groups (P > 0.05). None of the control group had history of alcohol abuse or a history of illicit substance use before or during their service. Twenty-seven draftees in the study group (11.2%) had a history of alcohol abuse and/or illicit substance use during their service and 23 of these had already abused alcohol and/or used illicit substances before service. Seventy-five draftees (49.3%) in the study group had sought help from counselors or psychiatrists before their service and all had sought help from outpatient psychiatric services during their service. After careful evaluation in the outpatient clinic, 64 (42.1%) were admitted to the psychiatric ward for further therapy as a result of acute and risky psychiatric syndromes.

**Table 2:** Demographics (N = 304)

	**Study Group**	**Control Group**	**P-value**
	**(n = 152)**	**(n = 152)**	
Age (years)	20.4 ± 4.2	20.2 ± 4.6	0.692
Education (years)	11.4 ± 2.1	11.6 ± 3.5	0.546
Marriage			0.890
Married	17 (11.2%)	19 (12.5%)	
Unmarried	135 (88.8%)	133 (87.5%)	
Service distribution			1.000
Army	64 (42.1%)	64 (42.1%)	
Navy	38 (25%)	38 (25%)	
Air Force	19 (12.5%)	19 (12.5%)	
Military Police	14 (9.2%)	14 (9.2%)	
Coast Guard	17 (11.2%)	17 (11.2%)	
Rank distribution			1.000
Private first class	24 (15.8%)	24 (15.8%)	
Private	41 (27%)	41 (27%)	
Recruit	87 (57.2%)	87 (57.2%)	
History of abuse of alcohol only			
Before the service	8 (5.3%)	0	
During the service	12 (7.9%)	0	
History of illicit substance abuse only			
Before the service	4 (2.6%)	0	
During the service	4 (2.6%)	0	
Both abuse of alcohol and illicit substance			
Before the service	11 (7.2%)	0	
During the service	11 (7.2%)	0	
Seeking psychiatric outpatient treatment			
Before the service	75 (49.3%)	0	
During the service	152 (100%)	0	
Admitted for mental disorder during service	64 (42.1%)	0	

Suicidal and homicidal ideations, plans, and attempts are shown in Table 3. Before service, the rates of suicidal ideation (23.7% vs. 3.9%, P < 0.001), plans (11.2% vs. 0.0%, P < 0.001) and attempted suicide (4.6% vs. 0.0%, P = 0.015) in the study group were significantly higher than those in the control group. The rate of homicidal ideation before service in the study group was also significantly higher than that in the control group (15.1% vs. 5.9%, P = 0.009).

**Table 3:** Suicidal and homicidal ideation, plans and attempts

	**Study Group**	**Control Group**	**P-value**
	**(n = 152)**	**(n = 152)**	
**Suicide**			
Before service			
Ideation	36(23.7%)	6(3.9%)	<0.001*
Plan	17(11.2%)	0.0(0%)	<0.001*
Attempt	7(4.6%)	0.0(0%)	0.015*
**Homicide**			
Before service			
Ideation	23(15.1%)	9(5.9%)	0.009*
Plan	11(7.2%)	10(6.6%)	0.821
Attempt	4(2.6%)	1(0.01%)	0.371
During the service			
Ideation	13(8.6%)	10(6.6%)	0.515
Plan	9(5.9%)	4(2.6%)	0.156
Attempt	2(1.3%)	0(0.0%)	0.498

There were significant differences in willingness to serve in Penghu before and during military service between the two groups (both P < 0.001). Half the subjects (76/152, 50%) in the study group had no or low willingness to serve in Penghu before service and 74.3% had no or low willingness to serve in Penghu after assignment there (Table 4). A total of 34 draftees in the control group (34/152, 22.4%) had no or low willingness to serve in Penghu before service. However, the number of draftees with no or low willingness increased to 50.7% (77/152) during t service there.

**Table 4:** Willingness to serve, ideation, plans and attempts to be away without official leave and willingness to return after leaving camp

	**Study Group**	**Control Group**	**P-value**
	**(n = 152)**	**(n = 152)**	
Willingness to serve			
Before service			<0.001*
No/Low	76(50%)	34(22.4%)	
Medium	53(34.9%)	63(41.4%)	
High/Very high	23(15.1%)	55(36.20%)	
During service			<0..001*
No/Low	113(74.3%)	77(50.7%)	
Medium	34(22.4%)	46(30.3%)	
High/Very high	5(3.3%)	29(19.1%)	
Risk of absence without official leave			
Ideation	48(31.6%)	26(17.1%)	0.003*
Plans	23(15.1%)	13(8.6%)	0.076
Attempts	7(4.6%)	0(0.0%)	0.015*
Willingness to return to camp			<0.001*
No/Low	92(60.5%)	33(21.7%)	
Medium	35(23.0%)	85(55.9%)	
High/Very high	25(16.4%)	34(22.4%)	

The risk of AWOL in the study group was significantly higher than that in the control group (Table 4). The rates of AWOL ideation (31.6% vs. 17.1%, P < 0.001) and attempted AWOL (4.6% vs. 0.0%, P = 0.015) in the study group were significantly higher than those in the control group. A total of 92 draftees (92/152, 60.5%) had low willingness to return to camp after vacation. Draftees in the control group had a significantly higher willingness to return to camp.

The families of 92 cases (60.5%) of those suspended from service for mental disorders blamed the military, believing that the stresses in the military or improper discipline were the major cause. Only 21 families (13.8%) believed that military life had no direct connection with the result. The other 39 families (25.7%) had no comment regarding the reasons for suspension.

## Discussion

In this study, we demonstrated that adjustment disorders did exist among Armed Forces draftees (AFD) in the Penghu Area, a high readiness military zone in the Taiwan Strait. In a group of the draftees who were service suspended for mental disorders (study group), the number of draftees seeking psychiatric outpatient treatment increased from 49.3% before service to 100% during the service. The number of draftees with no or low willingness to serve increased from 50% before service to 74.3% during the service. Even in control group, the draftees without mental disorder, the number of draftees with no or low willingness to serve increased from 22.4% before service to 50.7% during t service. In addition, adjustment disorders in the study group were more severe than in the control group in terms of higher rates of suicidal ideation, plans, attempted suicide, and homicidal ideation. The percentage of draftees with no or low willingness to serve in Penghu and the risk of AWOL during military service were also significantly different between the study group and the control group. Most families (60.5%) blamed the military for the onset of mental disorders in the draftees, while only 13.8% did not. This showed that most families had little knowledge or information about the fact that most mental disorders are multi-factorial in etiology and the pressures of t military life may be only partially responsible for draftee illnesses. Most family members hoped that the patients could be referred back to Taiwan for more comprehensive diagnosis and more advanced treatment. They did not think that the patients could get enough support from the medical service on Penghu Island. Because this was a high readiness zone, commanders of military units were usually reluctant to refer these patients back to a general hospital in Taiwan for fear that they would be investigated by a superior officer for the cause of the mental disorder. Some family members complained that the psychiatric disorders must have shown some obvious signs before service and their child or brother should have been diagnosed and precluded from conscription. More psycho-education about this issue is needed by both the families and the draftees.

Major depressive disorder as an Axis I diagnosis in DSM-IV was the most prevalent mental disorder for the study group during their military service. A similar result was found in the US army. According to a report by the US Department of Defense (USDOD) in 1998, of 1138 new USAF recruits who had a psychiatric evaluation before receiving any training, 349 were discharged for mental disorders. Results of the evaluation indicated that depressive disorders were the most common problems. Other common diagnoses in descending order included adjustment disorder (20%), post-traumatic stress disorder (PSTD) (19%), alcohol abuse or dependence (5%), and attention-deficit/hyperactivity disorder (5%) [4]. A study in Poland found that neurotic disorders were responsible for more than 50% of discharges from military service [5].

A relatively high percentage of cases were suspended from service for DSM Axis II diagnoses, as 29 cases (19.1%) were diagnosed as personality disorders (primarily antisocial and/or borderline disorders). Data from another military population in the US also indicated that adjustment disorders and personality disorders were the most common military mental problems [6]. Studies have shown that individuals suffering from these disorders find it more difficult to adjust to military life when they face special stresses [7]. The characteristics of these personality disorders, such as a lack of guilt often create serious trouble for the individual and the unit [8]. We can thus infer that adjustment disorders are very likely to occur in individuals suffering from personality disorders under the special situational stress of military life. As a result, the immature defense mechanisms and inappropriate personality traits of these individuals might be problematic for the military. This is particularly true on Penghu where draftees undoubtedly face harsher military training and suffer greater psychological stress that draftees in military zones elsewhere in Taiwan either in ordinary times or times of war readiness.

In Taiwan, draftees with an IQ below 85 on the WAIS-III-R standard IQ test or other evidences of limited intelligence such as poor performance, diminished learning ability, or poor daily life functioning as reported by family, superior officers or comrades may be considered for service suspension. In our study, 13 cases (8.6%) with borderline intellectual functioning (IQ between 70 and 84 on the WAIS-III-R) and 7 cases (4.6%) with mild mental retardation (IQ ranged from 50 or 55 and 69 on the WAIS-III-R) suspended their service after IQ testing, a review of their junior high school and elementary school academic records, and meticulous clinical observation. This study revealed that adjustment disorders were common co-morbid conditions found in all 20 draftees applying for service suspension because of insufficient intellectual functioning, just as they were in those individuals with personality disorders. The former may be the result of barriers in interpersonal skills and lack of social experience due to an inadequate ability to learn compounded by the stress of military life. In our study, it was surprising to find that 65.0% of those with impaired intellectual function (13 of 20) had completed senior high or senior high vocational school, one graduated from a 2-year senior college, and only 6 completed just junior high or elementary school. This suggests that comprehensive intelligence testing should be part of military recruiting.

Alcohol and drug abuse have long been major concerns for the USDOD. One report indicated that one out of 5 active US military personnel drank to excess [9]. A similar study in the UK found that 67% of men and 49% of women in the regular UK Armed Forces were engaged in “hazardous drinking” [10,11]. In our study, 27 draftees with service suspension had problems with alcohol and/or illicit drug abuse during their service. All but four of these had co-morbid personality disorders (data not shown). Of these, over 85% (23/27) already had a history of alcoholism and/or drug abuse before call-up; however, no intensive study on alcohol or drug abuse has been conducted on military personnel in Taiwan and neither alcohol nor drug addiction has been included as a reason for service suspension.

We found that the study group of conscripted soldiers who were suspended from service due to psychiatric disorders had significantly more psychiatric service utilization before conscription as 49.3% sought outpatient psychiatric treatment. Some individuals with psychiatric disorders, such as intellectual insufficiency, major depressive disorder, personality disorders, or schizophrenia must have shown obvious external signs, and should have been diagnosed and precluded from conscription in the selection process before entering the service. In addition, our study found that draftees with mental disorders based on both DSM Axis I and Axis II diagnoses during the service had a higher rate of suicidal ideation, plans, and attempts before service than did the control group. The rate of homicidal ideation before service in the study group was also higher than that in the control group. Those individuals with obvious symptoms should not be sent to a high-alert area, nor be allowed to enter military service. Conscription is the major source of troop strength in Taiwan so that the determination of service suspension for mental disorders is harsher than that in other countries. Although dysthymic disorder, depressive disorder not otherwise specified, alcohol abuse or dependency, and attention-deficit/hyperactivity disorder are psychiatric diagnoses not suitable for service according to the USAF [8], none of these qualify for service suspension or retirement in Taiwan. Due to the special military environment and the military threats from Mainland China, draftees in Taiwan are unable to suspend their service for minor mental disorders. The USDOD surveys all recruits for a history of depression and suicide attempts [8]. Young men called for military duty in Finland are subject to psychiatric evaluation [12]. Another Finnish study indicated that screening for disadvantaged childhood living conditions, unemployment, financial problems, and homelessness as well as mental problems identified conscripts with a higher probability of service suspension [13]. Such an approach should be considered in Taiwan. Better screening before conscription would avoid the waste of human resources, the costs of conscription, and the potential danger to these individuals and their fellow soldiers.

Some experimental rehabilitation programs are available in the UK and the USA [6,11] Such non-hospital interventions established by the military can effectively reduce the “premature discharge rate” and help military personnel with mental disorders or suicide attempts regain their original social and role functioning. Currently in Taiwan, the military has established “rescue and rehabilitation centers” in some areas to accept and treat draftees suffering from minor mental disorders which are not severe enough for service suspension. Such a practice should be further encouraged in order to promote the mental health of those charged with being in the first line of defense to protect their country.

There are limitations to this study. We were unable to collect data concerning psychological status before military service (before the draft) because the confidentiality of medical records is protected by law in Taiwan, so we might not be able to conclude that mental disorder was the result of heightened tension and stress in a high military alert zone. We were also unable to obtain clinical histories from the families of draftees regarding prior psychiatric disorders. We could not obtain medical information about the draftees after discharge so there is no follow-up information.

## Conclusions

In conclusion, the study group of conscripted soldiers who were suspended from service due to psychiatric disorders had significantly more psychiatric service utilization and a higher rate of suicidal ideation, plans, and attempts before service. In addition, adjustment disorders in the study group were more severe than those in the control group. The percentage of draftees who had low willingness to serve in Penghu and the risk of AWOL in the study group were also higher than in the control group. We therefore suggest close monitoring of draftees at risk for mental disorders at an early stage of military service to promote higher quality of troops in a high readiness military zone.

## Competing interests

The authors declare that they have no competing interests.

## Authors’ contributions

CWC and CKC conceived the research idea, prepared the instrument (Basic Data Survey questionnaire), recruited the participants, conducted the survey, and collected and analyzed the data. CHK helped conceive the idea and design of the research and prepared the manuscript. Both CHP and WHW helped recruit the participants and conduct the survey. All authors read and approved the final manuscript.

## Pre-publication history

The pre-publication history for this paper can be accessed here:

http://www.biomedcentral.com/1471-244X/12/46/prepub
